# Are multimorbidity patterns associated with fear of falling in community-dwelling older adults?

**DOI:** 10.1186/s12877-022-02889-9

**Published:** 2022-03-10

**Authors:** Jaquelini Betta Canever, Bruno de Souza Moreira, Ana Lúcia Danielewicz, Núbia Carelli Pereira de Avelar

**Affiliations:** 1 Laboratory of Aging, Resources and Rheumatology, Department of Health Sciences, Federal University of Santa Catarina, Campus Araranguá, Rod. Governador Jorge Lacerda, Araranguá, Santa Catarina, Urussanguinha 320188906-072 Brazil; 2 Fundação Oswaldo Cruz (Fiocruz), Belo Horizonte, Minas Gerais, Brazil

**Keywords:** Accidental Falls, Aged, Fear of Falling, Independent Living, Risk Factors

## Abstract

**Background:**

Multimorbidity is defined as the co-occurrence of multiple chronic or acute diseases and medical conditions in the same individual and can be grouped into different patterns based on the type of disease. These patterns are associated with poorer quality of life and premature death. It is believed that these patterns entail functional limitations, which may contribute to the fear of falling; however, this association remains unknown. Identifying this possible association is fundamental for developing individual and collective care approaches aimed at preventing the different patterns of chronic diseases in older adults in order to decrease the fear of falling. The objective of this study was to investigate the association between multimorbidity patterns and fear of falling in older adults.

**Methods:**

This was a cross-sectional study including 308 older adults. The exposure variables were the presence of three multimorbidity patterns (cardiopulmonary, musculoskeletal, and vascular-metabolic) and pattern association assessed by self-report of two or more similar coexisting chronic diseases. The outcome was fear of falling assessed by the Brazilian version of Falls Efficacy Scale-International (cut-off point ≥ 23 points). Multivariable logistic regression was used to analyze the association between variables.

**Results:**

Older adults with cardiopulmonary, musculoskeletal, vascular-metabolic patterns and pattern association had 3.49 (95%CI 1.13; 10.78), 2.03 (95%CI 1.13; 3.64), 2.14 (95%CI 1.20; 3.82), and 4.84 (95%CI 2.19; 10.68), respectively, greater chances of presenting fear of falling when compared to older adults without the patterns.

**Conclusions:**

The presence of multimorbidity patterns is associated with higher chances of reporting fear of falling. It is emphasized that the introduction of public health programs aimed at preventing multimorbidity patterns is essential to reduce possible adverse health outcomes, including fear of falling and its negative consequences for older adult health.

## Background

Falls are the leading cause of death in older adults, representing a serious public health problem [1, 2]. The occurrence of falls in older adults negatively influences their quality of life, leading to disabilities in activities of daily living [3], predisposes them to develop anxiety and depression [4], reduces body balance [5], and contributes to developing fear of falling [6]. Fear of falling can be defined as an exacerbated concern about falling during daily activities [7], which shows high prevalence among community-dwelling older adults, being observed in 41.7% of Spanish older adults [8], 75.6% of Korean older adults [9], and 48.4% of Brazilian older adults [10].

Fear of falling is related to adverse health events such as increased depressive symptoms [11], reduction in physical activity [5], functional decline [12], and increased risk of falls [13]. Several conditions can predispose a person to fear of falling, among which are female sex [14, 15], a negative health self-perception [16, 17], environmental factors such as residing near garbage accumulation and/or open sewers and high crime rates [10], the presence of cognitive decline [4, 7], and multimorbidity [8, 15].

Multimorbidity can be defined as the coexistence of two or more chronic or acute diseases of multifactorial nature, limited to different physiological systems and associated with negative health outcomes, increased disability and decreased quality of life [18]. In this context, the influence of different patterns of diseases related to multimorbidity on negative outcomes in older adults has been investigated, which are usually classified into cardiopulmonary, musculoskeletal, and vascular-metabolic patterns [19]. Evidence shows that older adults with multimorbidity patterns have a greater chance of functional disability [20, 21], dementia [22], institutionalization [23], and mortality [24].

It is noteworthy that although no studies have been found so far that relate the different multimorbidity patterns with fear of falling in older adults, there are studies that have demonstrated the association of isolated diseases with this outcome [25–30]. According to these studies, fear of falling is associated with heart disease [25, 30] (cardiopulmonary pattern), joint diseases [29, 31] (musculoskeletal pattern), and obesity, diabetes mellitus, and systemic arterial hypertension [26–28] (vascular-metabolic pattern). However, there is no evidence whether the concomitant presence of diseases that have clinical and physiological manifestations in the same body system also negatively interferes with fear of falling.

Thus, considering all the negative repercussions of the fear of falling together with the high prevalence of chronic diseases in older adults, it is essential to study the different multimorbidity involvement patterns and theirs relationship with the fear of falling. It is believed that these results can help in screening and early identification of older adults who fit into multimorbidity patterns with greater associations with fear of falling, providing a choice of better strategies to prevent and cope with fear of falling in older adults. Thus, the present study aimed to investigate the association between multimorbidity patterns and fear of falling in community-dwelling older adults.

## Methods

### Study design

This was a cross-sectional study with a probabilistic sample, carried out with older adults (60 years or older) of both sexes, registered in the health information system of Primary Care in the municipality of Balneário Arroio do Silva, Santa Catarina State, Brazil.

### Population and sample

The sample size calculation took into account the total older adult population registered in the municipality’s Health System (*n* = 2,833). The unknown prevalence for the outcomes of 50%, confidence level of 95%, and sampling error of six percentage points were considered for the sample calculation, thus estimating the need for 302 volunteers for the study. Foreseeing possible sample losses, 540 older adult subjects were eligible to compose the sample.

The study inclusion criteria were men and women aged 60 years or older living in the urban area of the municipality of Balneário Arroio do Silva, Santa Catarina State, Brazil. Older adults bedridden, dependent, or unable to answer the questionnaires, those living in long-term care institutions, and those who had changed their residential address were excluded. Older adults who were not located at their homes after three attempts made on different days and times were considered losses, while those who refused to participate in the study were considered refusals.

The older adult participants received guidance on the research objectives and signed an informed consent form. This study was approved by the Ethics Committee on Human Research of the Universidade Federal de Santa Catarina (CAAE no.87776318.3.0000.0121).

### Data collection procedure

Data collection was conducted between the months of September 2018 and September 2019. The selected older adults were initially contacted by phone and invited to participate in the study, and then visits to their homes were scheduled. All interviewers were previously trained to standardize the evaluation methods of the objective measures to be performed.

### Outcome variable

Fear of falling was assessed by the Falls Efficacy Scale-International-Brazil (FES-I-Brazil) [32], which was previously translated and adapted by Camargos et al*.* (2010) for use in Brazilian older adults. This scale evaluates the concern about suffering falls when performing 16 daily life tasks including simple activities such as dressing, undressing, and bathing, to more complex activities such as walking on uneven surfaces, going up or down stairs, and walking on slippery surfaces. The evaluated older adults were asked about their concerns on the possibility of falling when performing 16 activities. For each task, the score ranges from 1 to 4, and the total scale score can vary from 16 to 64 points [33]. The cut-off point established to discriminate high fear of falling in older adults in Brazil is a score equal to or greater than 23 [33]. This scale is a valid instrument to assess fear of falling in older adults with and without cognitive decline [34].

### Exposure variables

The independent variables were the presence of three multimorbidity patterns considering the self-reported occurrence of two or more diseases with similar clinical characteristics [35, 36]. The participants were asked the following question: "A doctor or health professional has already said that you have/had the following diseases: chronic bronchitis or asthma, cardiac diseases, and tuberculosis (cardiopulmonary pattern) [36, 37]; arthritis or rheumatism, chronic back problems, and osteoporosis (musculoskeletal pattern) [36, 38]; systemic arterial hypertension, diabetes mellitus, stroke, cancer, and chronic renal failure (vascular-metabolic pattern)?” [35, 39]. The pattern association (i.e. having two or three patterns) was also analyzed in this study [40–42].

### Adjustment variables

The following adjustment variables were used for this analysis: sex (female, male) [43]; age group (60–69 years, 70–79 years, ≥ 80 years); cognitive impairment assessed by the Mini-Mental State Examination (MMSE), translated and validated for Brazilian population [44]. The cut-off points for the classification of cognitive decline were proposed by Brucki et al. (2003) [45]: 20 points for illiterates; 25 points for people with 1 to 4 years of schooling; 26.5 points for those with 5 to 8 years of schooling; 28 points for those with 9 to 11 years of schooling, and 29 points for those with more than 11 years of schooling [8]; and sedentary behavior [< 248.57 min (low), ≥ 248.57 min (high) in sitting, lying down or reclining position] [46], as evaluated by the long version of the International Physical Activity Questionnaire (IPAQ), previously validated for the Brazilian population [47].

### Statistical analysis

The data were independently tabulated by two researchers in the Microsoft Excel software program (2019), and were subsequently entered into the SPSS database (IBM®, Chicago, IL, USA), version 23.0. The significance level adopted was 5%. Descriptive analyses were performed using absolute values and proportions (%). Crude and adjusted logistic regression analyses were performed to investigate the association between different multimorbidity patterns and fear of falling, estimating the odds ratio (OR) and their respective 95% confidence intervals (95%CI).

## Results

A total of 308 community-dwelling older adults (69.67 ± 6.99 years) participated in the study. The sample was predominantly composed of women (57.8%), aged 60–69 years (54.7%), without cognitive impairment (80.0%), and with low sedentary behavior (58.4%). The sample characterization is described in Table 1. The overall prevalence of fear of falling in the sample was 46.8%, with higher proportions among women (72.9%), aged 60–69 years (50.7%), without cognitive impairment (80.4%), and similar between low and high sedentary behavior groups (50.0%).

**Table 1:** Sociodemographic and lifestyle characteristics and multimorbidity patterns of community-dwelling older adults according to fear of falling (*n* = 308)

Variables	Total N (%)	Fear of falling	
		**Low fear of falling N (%)**	**High fear of falling N (%)**
Sex			
Female	178 (57.8)	73 (45.9)	102 (72.9)
Male	130 (42.2)	86 (54.1)	38 (27.1)
Age group (years)			
60–69	168 (54.7)	93 (58.9)	71 (50.7)
70–79	109 (35.5)	57 (36.1)	49 (35.0)
≥ 80	30 (9.8)	8 (5.1)	20 (6.7)
Cognitive impairment			
No	212 (80.0)	116 (80.0)	90 (80.4)
Yes	53 (20.0)	29 (20.0)	22 (19.6)
Sedentary behavior			
Low (< 248.57 min)	180 (58.4)	105 (66.0)	70 (50.0)
High (≥ 248.57 min)	128 (41.6)	54 (34.0)	70 (50.0)
Cardiopulmonary pattern			
Without pattern	288 (93.5)	154 (96.9)	126 (90.0)
With pattern	20 (6.5)	5 (3.1)	14 (10.0)
Musculoskeletal pattern			
Without pattern	200 (64.9)	121 (76.1)	72 (51.4)
With pattern	108 (35.1)	38 (23.9)	68 (48.6)
Vascular-metabolic pattern			
Without pattern	215 (70.0)	124 (78.0)	84 (60.4)
With pattern	92 (30.0)	35 (22.0)	55 (39.6)
Pattern association			
Without pattern	137 (44.6)	93 (58.5)	41 (29.5)
1 pattern	126 (41.0)	54 (34.0)	66 (47.5)
2 or 3 patterns	44 (14.3)	12 (7.5)	32 (23.0)

Legend: N: number; min: minutes.

As for the multimorbidity patterns, 6.5% had the cardiopulmonary pattern, 35.1% the musculoskeletal pattern, 30.0% the vascular-metabolic pattern and 14.3% of the participants had more than one pattern at the same time.

  The association between multimorbidity patterns and fear of falling is presented in Table 2. The adjusted logistic regression analysis showed that older adults with cardiopulmonary, musculoskeletal, vascular-metabolic patterns and pattern association had 3.49 (95%CI 1.13; 10.78), 2.03 (95%CI 1.13; 3.64), 2.14 (95%CI 1.20; 3.82), and 4.84 (95%CI 2.19; 10.68), respectively, greater chances of presenting fear of falling when compared to older adults without the patterns.

**Table 2:** Crude and adjusted logistic regression analyses between multimorbidity patterns and fear of falling in community-dwelling older adults

Variables	Fear of falling	
	**Crude** **OR (95%CI)**	**Adjustedª** **OR (95%CI)**
Cardiopulmonary pattern		
Without pattern	1.00	1.00
With pattern	3.24 (1.12; 9.37)	3.49 (1.13; 10.78)
Musculoskeletal pattern		
Without pattern	1.00	1.00
With pattern	2.70 (1.59; 4.61)	2.03 (1.13; 3.64)
Vascular-metabolic pattern		
Without pattern	1.00	1.00
With pattern	2.31 (1.34; 3.98)	2.14 (1.20; 3.82)
Pattern association		
Without pattern	1.00	1.00
1 pattern	2.85 (1.70; 4.78)	2.23 (1.29; 3.96)
2 or 3 patterns	6.13 (2.87; 13.12)	4.84 (2.19; 10.68)

ªAdjusted for the variables sex, age group, cognitive impairment, and sedentary behavior. Legend: OR: odds ratio; 95%CI: 95% confidence interval

## Discussion

This study showed that older adults with multimorbidity patterns were more likely to be afraid of falling when compared to those who did not have the same patterns, even after adjusting for sociodemographic and behavioral variables. It is noteworthy that the chances of presenting fear of falling were 3.5 times higher for older adults with cardiopulmonary diseases when compared to those who did not have the same involvement pattern.

Regarding the multimorbidity patterns, 6.5% of the older adults in this study presented a cardiopulmonary pattern, corroborating the findings of the National Health Survey conducted in Brazil in 2013, in which low prevalence was observed for this multimorbidity pattern (2.3%; 95%CI 2.0; 2.6) [35]. High prevalence rates of the cardiopulmonary pattern are observed in high population countries such as China (45.1%) and India (61.5%) due to the increase in chronic diseases such as COPD that occur due to high smoking and pollution rates [41]. A previous study has shown that older adults with COPD have a greater fear of falling (FES-I score > 25) than older adults without this condition (FES-I score < 20) [48]. This association can be explained by the fact that COPD patients have lower quadriceps femoris muscle strength, reduced physical activity, and balance deficits [48]. It is noteworthy that the cardiopulmonary pattern increased the chances of an older adult having fear of falling by 3.49 (95%CI 1.13; 10.78) in our study, corroborating other findings that demonstrated an association between diseases of the same pattern [25, 30]. Heart disease increased the chances of the older adults have fear of falling by 25% [25], which was associated with low confidence to perform physical activities and restricted activities of daily living and social activities; factors known to increase the fear of falling [30]. In addition, some conditions such as heart failure lead to reduced cardiac output and oxygen uptake in the muscle, which decreases aerobic capacity, leading the older adult to tire more easily and restrict their physical activities, thus increasing the fear of falling [49].

About 35.0% of the older adults in the current sample presented a musculoskeletal pattern; a lower prevalence compared with the findings of the study by Salazar et al. [50], in which the musculoskeletal pattern was present in 66% of Spanish adults. This difference in prevalence between studies may be because Salazar et al. [50] included individuals over 18 years in their sample and the determination of the prevalence of musculoskeletal conditions was not performed only for the older adult population. Moreover, older adults with a musculoskeletal pattern had 2.03 (95%CI 1.13; 3.64) higher chances of presenting fear of falling in the present study. This is due to the fact that this pattern is strongly related to rheumatic and joint diseases, which may increase the risk of older adults developing fear of falling [29, 35]. In addition, other studies show that musculoskeletal conditions such as pain and osteoporosis increase the chances of older adults presenting fear of falling by 1.76 (95%CI 1.02; 3.04) [51] and 2.04 (95%CI 1.60; 2.60) [52], respectively. According to the literature, disorders such as pain or chronic conditions would exacerbate the disabling effect of fear of falling, as well as reduce healthy behaviors and patient compliance to treatment [53]. Recently, Meyer et al. [52] found that older adults with musculoskeletal disorders had greater joint instability, lower bone mass, frailty, and body balance deficits, all of which contributed to increased fear of falling.

Thirty percent of  the older adults in the present sample presented a vascular-metabolic pattern, corroborating the findings in the study by Schmidt et al. [35], in which the prevalence for this pattern was 30.9% (95%CI 29.9; 31.9) for the Brazilian population. Furthermore, the vascular-metabolic pattern in the present study increased the chances of an older adult presenting fear of falling by 2.14 (95%CI 1.20; 3.82), also corroborating the findings which verified such association, but investigated diseases of this group separately [26, 27, 54, 55]. Neri et al. [56] demonstrated that obesity increased the chances of older adults having fear of falling by 30% (± 8.40), which was associated with a higher risk of tripping, slipping, and falling among obese individuals, who prefer to remain at rest due to fear of falling [57]. In addition, obese individuals present lower balance due to anteriorization of the body mass center and altered sensory functions derived from the high body mass index, which increases postural oscillations, and consequently the fear of falling in this population [58].

Kelly et al. [26] investigated the association between diabetes mellitus and fear of falling, and found that diabetic individuals presented an 82.2% greater probability of fear of falling. This finding was associated with the fact that individuals with diabetes mellitus have worse gait performance, and therefore greater concern about falling [59]. Another important finding concerns reduced skin sensitivity in diabetics, which results in insecurity when performing activities of daily living and consequently greater fear of falling. Reduced skin sensitivity, especially in the plantar region, might lead to the balance deficit, which in turn, might affect older adults' confidence in stepping on uneven and/or sharp places and result in falls and fear of falling [26, 60]. In a study by Chang et al. [28], 50.0% of community-dwelling older adults with systemic arterial hypertension presented fear of falling, which was associated with low muscle strength, especially in the lower limbs (quadriceps femoris). In addition, older adults with systemic arterial hypertension were more likely to have mental disorders (anxiety and depression) that contribute to social restriction and physical limitation, which in turn favor the emergence of fear of falling [28].

It is noteworthy that the cardiopulmonary pattern was the most associated with fear of falling in this study when compared to the other patterns. This finding may be associated with the fact that cardiorespiratory conditioning can be reduced in older adults due to the senescence process and aggravated by the sedentary lifestyle adopted by many older adults [61]. Cardiorespiratory fitness, also affected by chronic conditions prevalent in older adults, such as COPD and chronic heart failure [62], is an important regulator of cerebral blood flow during physical activities [63]. Thus, when older adults who have cardiopulmonary diseases perform some functional activity such as walking, they may feel pre-syncope or be unable to perform such task due to extreme fatigue, consequently increasing the fear of falling, as highlighted in the study by McCarthy et al. [64].

Although the musculoskeletal and vascular-metabolic patterns have associations of lower magnitude with fear of falling than the cardiopulmonary pattern, these patterns present considerable odds ratios (around 2). In relation to the musculoskeletal pattern, its association with fear of falling may be due to muscle weakness, pain [65], and reduction in balance and proprioception [66]. Regarding the vascular-metabolic pattern, studies state that diseases related to this pattern are associated with the occurrence of falls and fear of falling due to different pathophysiological processes such as the increase in inflammatory biomarkers, which can trigger functional decline, reduced muscle strength, and gait changes, well-known factors that culminate in the fear of falling [67–69].

The association of two or three patterns increased the chances of the older adults reporting fear of falling. It is noteworthy that this finding corroborates previous studies [38, 70] and might be related to the fact that the presence of associated conditions in different systems can increase negative health self-perception, and culminate in frailty [71]. Frailty is considered a geriatric syndrome associated with several adverse health outcomes, including the fear of falling [72].

The originality is the greatest strength of the present study. To the best of our knowledge, this is the first study that proposed to examine the association of different multimorbidity patterns with the fear of falling in community-dwelling older adults. Thus, our results may help in proposing collective actions to prevent fear of falling directed to older adults with different multimorbidity patterns. For clinical practice, our findings can contribute to health professionals to carry out promotion and education actions on the multimorbidity patterns in a more specific way, aiming to inform the older adults about the various negative health outcomes that fear of falling can cause.

One of the limitations of this study is the cross-sectional design, which is subject to reverse causality in the relationship between multimorbidity patterns and fear of falling. The fact that the sample, despite being probabilistic, was exclusively composed of older adult residents of the southernmost region of Santa Catarina State, Brazil, prevents extrapolating the results to populations of places with different sociodemographic and environmental characteristics. Finally, it is noteworthy that chronic diseases were collected through self-report, which may be subject to biases such as memory bias, thus preventing their clinical confirmation.

## Conclusion

Older adults with investigated multimorbidity patterns were more likely to have fear of falling compared to those who did not have the same patterns of diseases. It is noteworthy that public health programs aimed at preventing multimorbidity patterns are essential to reduce possible negative health outcomes related to fear of falling, especially falls that lead to functional limitations and increase the risk of death.

## Data Availability

The datasets used and/or analyzed during the current study are available from the corresponding author on reasonable request.

## References

[1] Kruschke C, Butcher HK (2017). Evidence-based practice guideline: Fall prevention for older adults. J Gerontol Nurs.

[2] Peel NM (2011). Epidemiology of falls in older age. Can J Aging.

[3] Smith A de A, Silva AO, Rodrigues RAP, Moreira MASP, Nogueira J de A, Tura LFR. Avaliação do risco de quedas em idosos residentes em domicílio. Revista Latino-Americana de Enfermagem. 2017;25. 10.1590/1518-8345.0671.2754 PMC5396481 28403333

[4] Akyol Y, Ulus Y, Tander B, Tomak L, Zahiroğlu Y, Bilgici A (2018). Falls, fear of falling, and associated factors in ambulatory patients with rheumatoid arthritis: A comparative study with healthy controls. Turk J Phys Med Rehabil.

[5] Lopes KT, Costa DF, Santos LF, Castro DP, Bastone AC (2009). Prevalência do medo de cair em uma população de idosos da comunidade e sua correlação com mobilidade, equilíbrio dinâmico, risco e histórico de quedas. Rev Bras Fisioter.

[6] Ang GC, Low SL, How CH (2020). Approach to falls among the elderly in the community. Singapore Med J.

[7] Jung D (2008). Fear of falling in older adults: comprehensive review. Asian Nurs Res.

[8] Lavedán A, Viladrosa M, Jürschik P, Botigué T, Nuín C, Masot O (2018). Fear of falling in community-dwelling older adults: A cause of falls, a consequence, or both?. PLoS ONE.

[9] Oh E, Hong GRS, Lee S, Han S (2017). Fear of falling and its predictors among community-living older adults in Korea. Aging Ment Health.

[10] Canever JB, Danielewicz AL, Leopoldino AAO, de Avelar NCP (2021). Is the self-perception of the built neighborhood associated with fear of falling in community-dwelling older adults ?. Arch Gerontol Geriatr.

[11] Hajek A, König H-H (2020). What are the psychosocial consequences when fear of falling starts or ends? Evidence from an asymmetric fixed effects analysis based on longitudinal data from the general population. Int J Geriatr Psychiatry.

[12] Auais M, French S, Alvarado B, Pirkle C, Belanger E, Guralnik J (2018). Fear of Falling Predicts Incidence of Functional Disability 2 Years Later: A Perspective From an International Cohort Study. J Gerontol A Biol Sci Med Sci.

[13] Young WR, Mark WA (2015). How fear of falling can increase fall-risk in older adults: Applying psychological theory to practical observations. Gait Posture.

[14] Vitorino LM, Marques-Vieira C, Low G, Sousa L, Cruz JP (2019). Fear of falling among Brazilian and Portuguese older adults. Int J Older People Nurs.

[15] Moreira B de S, Andrade AC de S, Xavier CC, Proietti FA, Braga L de S, Friche AA de L, et al. Perceived neighborhood and fall history among community-dwelling older adults living in a large Brazilian urban area: a multilevel approach. International Journal of Environmental Health Research. 2020;:1–13. 10.1080/09603123.2020.1782354. 10.1080/09603123.2020.1782354 32568556

[16] Vitorino LM, Teixeira CAB, Boas ELV, Pereira RL, dos Santos NO, Rozendo CA (2017). Fear of falling in older adults living at home: Associated factors. Revista da Escola de Enfermagem.

[17] Ferreira FR, César CC, de Andrade FB, de Souza Junior PRB, Lima-Costa MF, Proietti FA (2018). Aspects of social participation and neighborhood perception: ELSI-Brazil. Rev Saude Publica.

[18] Starfield B, Lemke KW, Bernhardt T, Foldes SS, Forrest CB, Weiner JP (2003). Comorbidity: implications for the importance of primary care in “case” management. Ann Fam Med.

[19] Prados-Torres A, Calderón-Larrañaga A, Hancco-Saavedra J, Poblador-Plou B, van den Akker M (2014). Multimorbidity patterns: a systematic review. J Clin Epidemiol.

[20] Vetrano DL, Rizzuto D, Calderón-Larrañaga A, Onder G, Welmer AK, Qiu C (2019). Walking Speed Drives the Prognosis of Older Adults with Cardiovascular and Neuropsychiatric Multimorbidity. Am J Med.

[21] Schmidt TP, Wagner K, Schneider I, Danielewicz A (2020). Multimorbidity patterns and functional disability in elderly Brazilians: a cross-sectional study with data from the Brazilian National Health Survey. Cad Saude Publica.

[22] Grande G, Marengoni A, Vetrano D, Roso-Llorach A, Rizzuto D, Zucchelli A (2021). Multimorbidity burden and dementia risk in older adults: The role of inflammation and genetics. Alzheimers Dement.

[23] Marengoni A, Akugizibwe R, Vetrano DL, Roso-Llorach A, Onder G, Welmer A-K (2021). Patterns of multimorbidity and risk of disability in community-dwelling older persons. Aging Clin Exp Res.

[24] Vetrano DL, Roso-Llorach A, Fernández S, G-C M, V C, O G (2020). Twelve-year clinical trajectories of multimorbidity in a population of older adults. Nat Commun.

[25] Aburub AS, Phillips SP, Aldughmi M, Curcio C-L, Guerra RO, Auais M. Fear of Falling Among Community-Dwelling Older Adults with Heart Disease: Findings from an International Mobility in Aging Study (IMIAS). Physiotherapy theory and practice. 2021;:1–14. 10.1080/09593985.2021.1901327 33726620

[26] Kelly C, Fleischer A, Yalla S, Grewal GS, Albright R, Berns D (2013). Fear of falling is prevalent in older adults with diabetes mellitus but is unrelated to level of neuropathy. J Am Podiatr Med Assoc.

[27] Rosic G, Milston AM, Richards J, Dey P (2019). Fear of falling in obese women under 50 years of age: a cross-sectional study with exploration of the relationship with physical activity. BMC obesity.

[28] Chang HT, Chen HC, Chou P (2016). Factors associated with fear of falling among community-dwelling older adults in the Shih-Pai Study in Taiwan. PLoS ONE.

[29] Canever JB, Danielewicz AL, Avelar NCP (2020). Associação entre aspectos físicos-funcionais, comportamentais e de saúde com o medo de cair em idosos comunitários. Acta Fisiátrica.

[30] Lapier TK, Cleary K, Kidd J (2009). Exercise self-efficacy, habitual physical activity, and fear of falling in patients with coronary heart disease. Cardiopulm Phys Ther J.

[31] Painter JA, Allison L, Dhingra P, Daughtery J, Cogdill K, Trujillo LG (2012). Fear of falling and its relationship with anxiety, depression, and activity engagement among community-dwelling older adults. Am J Occup Ther.

[32] Yardley L, Beyer N, Hauer K, Kempen G, Piot-Ziegler C, Todd C (2005). Development and initial validation of the Falls Efficacy Scale-International (FES-I). Age Ageing.

[33] Camargos FFO, Dias RC, Dias JMD, Freire MTF (2010). Cross-cultural adaptation and evaluation of the psychometric properties of the Falls Efficacy Scale-International Among Elderly Brazilians (FES-I-BRAZIL). Rev Bras Fisioter.

[34] Hauer KA, Kempen GIJM, Schwenk M, Yardley L, Beyer N, Todd C (2011). Validity and Sensitivity to Change of the Falls Efficacy Scales International to Assess Fear of Falling in Older Adults with and without Cognitive Impairment. Gerontology.

[35] Schmidt TP, Wagner KJP, Schneider IJC, Danielewicz AL (2020). Multimorbidity patterns and functional disability in elderly Brazilians: A cross-sectional study with data from the Brazilian National Health Survey. Cad Saude Publica.

[36] Baré M, Herranz S, Roso-Llorach A, Jordana R, Violán C, Lleal M (2021). Multimorbidity patterns of chronic conditions and geriatric syndromes in older patients from the MoPIM multicentre cohort study. BMJ open.

[37] Yao S-S, Cao G-Y, Li M, Ai P, Huang Z, Xu B (2018). The prevalence and patterns of multimorbidity among community-dwelling older adults in China: a cross-sectional study. The Lancet.

[38] Wang Z, Peng W, Li M, Li X, Yang T, Li C (2021). Association between multimorbidity patterns and disability among older people covered by long-term care insurance in Shanghai. China BMC Public Health.

[39] Yao S-S, Cao G-Y, Han L, Chen Z-S, Huang Z-T, Gong P (2020). Prevalence and Patterns of Multimorbidity in a Nationally Representative Sample of Older Chinese: Results From the China Health and Retirement Longitudinal Study. J Gerontol A Biol Sci Med Sci.

[40] Rivera-Almaraz A, Manrique-Espinoza B, Ávila-Funes JA, Chatterji S, Naidoo N, Kowal P (2018). Disability, quality of life and all-cause mortality in older Mexican adults: association with multimorbidity and frailty. BMC Geriatr.

[41] Garin N, Koyanagi A, Chatterji S, Tyrovolas S, Olaya B, Leonardi M (2016). Global Multimorbidity Patterns: A Cross-Sectional, Population-Based, Multi-Country Study. J Gerontol A Biol Sci Med Sci.

[42] Violan C, Foguet-Boreu Q, Flores-Mateo G, Salisbury C, Blom J, Freitag M (2014). Prevalence, determinants and patterns of multimorbidity in primary care: a systematic review of observational studies. PloS One.

[43] Schäfer I, Hansen H, Schön G, Höfels S, Altiner A, Dahlhaus A (2012). The influence of age, gender and socio-economic status on multimorbidity patterns in primary care. first results from the multicare cohort study. BMC Health Serv Res.

[44] Bertolucci PHF, Brucki S, Campacci SR, Juliano Y (1994). O mini-exame do estado mental em uma população geral: impacto da escolaridade. Arq Neuropsiquiatr.

[45] Brucki S, Nitrini R, Caramelli P, Bertolucci PHF, Okamoto IH (2003). Sugestões para o uso do mini-exame do estado mental no Brasil. Arq Neuropsiquiatr.

[46] Canever JB, Danielewicz AL, Leopoldino AAO, Giehl MWC, de Avelar NCP (2021). How Much Time in Sedentary Behavior Should Be Reduced to Decrease Fear of Falling and Falls in Community-Dwelling Older Adults?. J Aging Phys Act.

[47] Matsudo S, Araújo T, Matsudo V, Andrade D, Andrade E, Oliveira LC, et al. Questionário Internacional De Atividade Física (Ipaq): Estupo De Validade E Reprodutibilidade No Brasil. Questionário Internacional De Atividade Física (Ipaq): Estupo De Validade E Reprodutibilidade No Brasil 2001;6:5–18.

[48] Oliveira CC, McGinley J, Lee AL, Irving LB, Denehy L (2015). Fear of falling in people with chronic obstructive pulmonary disease. Respir Med.

[49] Manini TM, Pahor M (2009). Physical activity and maintaining physical function in older adults. Br J Sports Med.

[50] Salazar A, Dueñas M, Ojeda B, Failde I (2014). Association of painful musculoskeletal conditions and migraine headache with mental and sleep disorders among adults with disabilities, Spain, 2007–2008. Prev Chronic Dis.

[51] Mat S, Kamaruzzaman SB, Chin A-V, Tan MP (2020). Impact of Knee Pain on Fear of Falling, Changes in Instrumental Activities of Daily Living, and Falls Among Malaysians Age 55 Years and Above. Front Public Health.

[52] Meyer F, König HH, Hajek A. Osteoporosis, fear of falling, and restrictions in daily living. Evidence from a nationally representative sample of community-dwelling older adults. Frontiers in Endocrinology. 2019;10 SEP:1–6. 10.3389/fendo.2019.00646 PMC6775197 31616377

[53] Scott KM, Von Korff M, Alonso J, Angermeyer MC, Bromet E, Fayyad J (2009). Mental-physical co-morbidity and its relationship with disability: results from the World Mental Health Surveys. Psychol Med.

[54] Lee S, Oh E, Hong GRS (2018). Comparison of factors associated with fear of falling between older adults with and without a fall history. Int J Environ Res Public Health.

[55] Neri SGR, Gadelha AB, de David AC, Ferreira AP, Safons MP, Tiedemann A (2001). The Association Between Body Adiposity Measures, Postural Balance, Fear of Falling, and Fall Risk in Older Community-Dwelling Women. Journal of geriatric physical therapy.

[56] Neri SGR, Gadelha AB, Correia ALM, Pereira JC, Safons MP, Lima RM (2017). Association between obesity, risk of falls and fear of falling in older women. Revista Brasileira de Cineantropometria e Desempenho Humano.

[57] Yang F, Kim J, Yang F (2017). Effects of obesity on dynamic stability control during recovery from a treadmill-induced slip among young adults. J Biomech.

[58] Son SM (2016). Influence of Obesity on Postural Stability in Young Adults. Osong Public Health Res Perspect.

[59] Resnick HE, Vinik AI, Schwartz AV, Leveille SG, Brancati FL, Balfour J (2000). Independent effects of peripheral nerve dysfunction on lower-extremity physical function in old age: the Women’s Health and Aging Study. Diabetes Care.

[60] Bretan O, Pinheiro RM, Corrente JE (2010). Avaliação funcional do equilíbrio e da sensibilidade cutânea plantar de idosos moradores na comunidade. Braz J Otorhinolaryngol.

[61] Jackson AS, Sui X, Hébert JR, Church TS, Blair SN (2009). Role of lifestyle and aging on the longitudinal change in cardiorespiratory fitness. Arch Intern Med.

[62] He Z, Bian J, Carretta HJ, Lee J, Hogan WR, Shenkman E (2018). Prevalence of Multiple Chronic Conditions Among Older Adults in Florida and the United States: Comparative Analysis of the OneFlorida Data Trust and National Inpatient Sample. J Med Internet Res.

[63] Zimmerman B, Sutton BP, Low KA, Fletcher MA, Tan CH, Schneider-Garces N (2014). Cardiorespiratory fitness mediates the effects of aging on cerebral blood flow. Frontiers in aging neuroscience.

[64] McCarthy K, Ward M, Romero Ortuño R, Kenny RA (2020). Syncope, fear of falling and quality of life among older adults: findings from the irish longitudinal study on aging (TILDA). Frontiers in cardiovascular medicine.

[65] Stubbs B, West E, Patchay S, Schofield P (2014). Is there a relationship between pain and psychological concerns related to falling in community dwelling older adults?. A systematic review Disability and rehabilitation.

[66] Monteiro AM, Forte P, Carvalho J, Barbosa TM, Morais JE (2021). Relationship between fear of falling and balance factors in healthy elderly women: A confirmatory analysis. J Women Aging.

[67] Immonen M, Haapea M, Similä H, Enwald H, Keränen N, Kangas M (2020). Association between chronic diseases and falls among a sample of older people in Finland. BMC Geriatr.

[68] Paliwal Y, Slattum PW, Ratliff SM (2017). Chronic Health Conditions as a Risk Factor for Falls among the Community-Dwelling US Older Adults: A Zero-Inflated Regression Modeling Approach. Biomed Res Int.

[69] de Amorim JSC, Torres KCL, Teixeira-Carvalho A, Martins-Filho OA, Lima-Costa MF (2019). Peixoto SV.

[70] Lee Y, Kim H, Jeong H, Noh Y (2020). Patterns of Multimorbidity in Adults: An Association Rules Analysis Using the Korea Health Panel. Int J Environ Res Public Health.

[71] Villacampa-Fernández P, Navarro-Pardo E, Tarín JJ, Cano A (2017). Frailty and multimorbidity: Two related yet different concepts. Maturitas.

[72] Martínez-Arnau FM, Prieto-Contreras L, Pérez-Ros P (2021). Factors associated with fear of falling among frail older adults. Geriatric nursing (New York, NY).

